# Author Correction: Dissociating task acquisition from expression during learning reveals latent knowledge

**DOI:** 10.1038/s41467-020-17023-9

**Published:** 2020-06-18

**Authors:** Kishore V. Kuchibhotla, Tom Hindmarsh Sten, Eleni S. Papadoyannis, Sarah Elnozahy, Kelly A. Fogelson, Rupesh Kumar, Yves Boubenec, Peter C. Holland, Srdjan Ostojic, Robert C. Froemke

**Affiliations:** 1 Department of Psychological and Brain Sciences, Johns Hopkins University, Baltimore, MD 21218 USA; 2 Department of Neuroscience, Johns Hopkins Medical School, Baltimore, MD 21218 USA; 3 Departments of Otolaryngology, Neuroscience and Physiology, Skirball Institute, Neuroscience Institute, New York University School of Medicine, New York, NY 10016 USA; 4 Center for Neural Science, New York University, New York, NY 10003 USA; 5 Laboratoire des Systèmes Perceptifs, UMR8248, École Normale Supérieure-PSL Research University, 75006 Paris, France; 6 Laboratoire de Neurosciences Cognitives, INSERM U960, École Normale Supérieure-PSL Research University, 75006 Paris, France; 7 Faculty Scholar, Howard Hughes Medical Institute, Chevy Chase, MA 20815 USA; 8 Present Address: Laboratory of Neurophysiology and Behavior, The Rockefeller University, New York, NY 10065 USA

**Keywords:** Learning and memory, Decision

Correction to: *Nature Communications* 10.1038/s41467-019-10089-0, published online 14 May 2019.

The original version of this Article omitted the following from the Acknowledgements.

HopkinsPREP NIH training program (R25 GM109441) (K.A.F.).

This has now been corrected in both the PDF and HTML versions of the Article.

