# Supplementary material for: Dissociating task acquisition from expression during learning reveals latent knowledge
Source: Nat Commun. 2019 May 14;10:2151. doi: 10.1038/s41467-019-10089-0 (PMC6517418; doi:10.1038/s41467-019-10089-0)
Supplement: Supplementary file 3 — Reporting Summary [file 41467_2019_10089_MOESM3_ESM.pdf]

nature research

Corresponding author(s): Kishore V. Kuchibhotla

Last updated by author(s): Apr 18, 2019

Reporting Summary

Nature Research wishes to improve the reproducibility of the work that we publish. This form provides structure for consistency and transparency in reporting. For further information on Nature Research policies, see Authors & Referees and the Editorial Policy Checklist .

Statistics

For all statistical analyses, confirm that the following items are present in the figure legend, table legend, main text, or Methods section.

n/a	Confirmed	
☐	☒	The exact sample size (*n*) for each experimental group/condition, given as a discrete number and unit of measurement
☐	☒	A statement on whether measurements were taken from distinct samples or whether the same sample was measured repeatedly
☐	☒	The statistical test(s) used AND whether they are one- or two-sided<br>*Only common tests should be described solely by name; describe more complex techniques in the Methods section.*
☐	☒	A description of all covariates tested
☐	☒	A description of any assumptions or corrections, such as tests of normality and adjustment for multiple comparisons
☐	☒	A full description of the statistical parameters including central tendency (e.g. means) or other basic estimates (e.g. regression coefficient) AND variation (e.g. standard deviation) or associated estimates of uncertainty (e.g. confidence intervals)
☐	☒	For null hypothesis testing, the test statistic (e.g. *F*, *t*, *r*) with confidence intervals, effect sizes, degrees of freedom and *P* value noted<br>*Give P values as exact values whenever suitable.*
☐	☒	For Bayesian analysis, information on the choice of priors and Markov chain Monte Carlo settings
☐	☒	For hierarchical and complex designs, identification of the appropriate level for tests and full reporting of outcomes
☐	☒	Estimates of effect sizes (e.g. Cohen's *d*, Pearson's *r*), indicating how they were calculated

Our web collection on statistics for biologists contains articles on many of the points above.

Software and code

Policy information about availability of computer code

Data collection	All behavioral data was collected using custom-written programs to control behavioral parameters.
Data analysis	We used MATLAB (R2016a) for analysis and GraphPad Prism 7 for statistics and data visualization. Custom MATLAB code was written for a theoretical model which has been made available to editors and reviewers

For manuscripts utilizing custom algorithms or software that are central to the research but not yet described in published literature, software must be made available to editors/reviewers. We strongly encourage code deposition in a community repository (e.g. GitHub). See the Nature Research guidelines for submitting code & software for further information.

Data

Policy information about availability of data

All manuscripts must include a data availability statement. This statement should provide the following information, where applicable:

- Accession codes, unique identifiers, or web links for publicly available datasets
- A list of figures that have associated raw data
- A description of any restrictions on data availability

All behavioral data that underlies the findings of this study, as well as all code related to the modeling work, is available at: http://froemkelab.med.nyu.edu/sites/default/files/Data_NatComms.zip

Field-specific reporting

Please select the one below that is the best fit for your research. If you are not sure, read the appropriate sections before making your selection.

Life sciences study design

All studies must disclose on these points even when the disclosure is negative.

Sample size	Sample sizes were determined to be appropriate based on the large effect size and consistency across animals.
Data exclusions	No animals were excluded from this analysis; in certain analyses, a subset of animals was used due to the behavioral testing frequency. During model fitting we excluded a small number of training blocks during which the animals executed little to no behavioral responses (two training blocks total across seven animals) or because of insufficient number of trials (one training block in which there was only 10 trials).
Replication	All attempts at replication were successful.
Randomization	This is not relevant to the study as behavior was a quantitative assessment with no "treatment" groups.
Blinding	Blinding was not relevant for this study as behavior was assessed quantitatively based on objective, measured criteria.

Reporting for specific materials, systems and methods

We require information from authors about some types of materials, experimental systems and methods used in many studies. Here, indicate whether each material, system or method listed is relevant to your study. If you are not sure if a list item applies to your research, read the appropriate section before selecting a response.

Materials & experimental systems

n/a	Involved in the study	
☒	☐	Antibodies
☒	☐	Eukaryotic cell lines
☒	☐	Palaeontology
☐	☒	Animals and other organisms
☒	☐	Human research participants
☒	☐	Clinical data

Methods

n/a	Involved in the study	
☒	☐	ChIP-seq
☒	☐	Flow cytometry
☒	☐	MRI-based neuroimaging

Animals and other organisms

Policy information about studies involving animals; ARRIVE guidelines recommended for reporting animal research

Laboratory animals	Mice of mixed sex were used at 8-16 weeks of age. Multiple strains were used (C57/BL6, PV-cre, ChAT-ChR2). Long-evans rats of approximately 90 days of age were used for rat studies. All ferret studies conformed to standards specified and approved by the French Ministry of Research and the ethics committee.
Wild animals	*Provide details on animals observed in or captured in the field; report species, sex and age where possible. Describe how animals were caught and transported and what happened to captive animals after the study (if killed, explain why and describe method; if released, say where and when) OR state that the study did not involve wild animals.*
Field-collected samples	*For laboratory work with field-collected samples, describe all relevant parameters such as housing, maintenance, temperature, photoperiod and end-of-experiment protocol OR state that the study did not involve samples collected from the field.*
Ethics oversight	JHU, NYU, Duke IACUC protocols, French Ministry of Research protocol

Note that full information on the approval of the study protocol must also be provided in the manuscript.
