# Supplementary material for: Dissociating task acquisition from expression during learning reveals latent knowledge
Source: Nat Commun. 2019 May 14;10:2151. doi: 10.1038/s41467-019-10089-0 (PMC6517418; doi:10.1038/s41467-019-10089-0)
Supplement: Supplementary file 4 — Description of Additional Supplementary Files [file 41467_2019_10089_MOESM4_ESM.pdf]

Description of Additional Supplementary Files

File Name: Supplementary Movie 1
Description: Mouse performing at expert levels in the reinforced context after prolonged training.

File Name: Supplementary Movie 2
Description: Video of mouse behaving in the reinforced context (trial block 1500-2000).

File Name: Supplementary Movie 3
Description: Video of mouse behaving in the probe context (trial block 1500-2000, immediately after Supplementary Movie 2).

File Name: Supplementary Movie 4
Description: Video of mouse behaving in the reinforced context with lever (trial block 1500-2000).

File Name: Supplementary Movie 5
Description: Video of mouse behaving in the probe context with (trial block 1500-2000, immediately after Supplementary Movie 4).
